# Linking CRISPR-Cas9 interference in cassava to the evolution of editing-resistant geminiviruses

**DOI:** 10.1186/s13059-019-1678-3

**Published:** 2019-04-25

**Authors:** Devang Mehta, Alessandra Stürchler, Ravi B. Anjanappa, Syed Shan-e-Ali Zaidi, Matthias Hirsch-Hoffmann, Wilhelm Gruissem, Hervé Vanderschuren

**Affiliations:** 1 Laboratory of Plant Biotechnology, Institute of Molecular Plant Biology, Department of Biology, ETH Zurich, Universitätstrasse 2, 8092 Zurich, Switzerland; 2 Laboratory of Plant Genomics, Department of Biological Sciences, University of Alberta, Edmonton, AB T6G 2E9 Canada; 3 Plant Genetics Lab, TERRA Research and Teaching Centre, Gembloux Agro BioTech, University of Liège, Passage des Déportés 2, 5030 Gembloux, Belgium

**Keywords:** Geminivirus, CRISPR-Cas9, Plant immunity, Genetic engineering, Cassava, ACMV

## Abstract

**Background:**

Geminiviruses cause damaging diseases in several important crop species. However, limited progress has been made in developing crop varieties resistant to these highly diverse DNA viruses. Recently, the bacterial CRISPR/Cas9 system has been transferred to plants to target and confer immunity to geminiviruses. In this study, we use CRISPR-Cas9 interference in the staple food crop cassava with the aim of engineering resistance to African cassava mosaic virus, a member of a widespread and important family (Geminiviridae) of plant-pathogenic DNA viruses.

**Results:**

Our results show that the CRISPR system fails to confer effective resistance to the virus during glasshouse inoculations. Further, we find that between 33 and 48% of edited virus genomes evolve a conserved single-nucleotide mutation that confers resistance to CRISPR-Cas9 cleavage. We also find that in the model plant *Nicotiana benthamiana* the replication of the novel, mutant virus is dependent on the presence of the wild-type virus.

**Conclusions:**

Our study highlights the risks associated with CRISPR-Cas9 virus immunity in eukaryotes given that the mutagenic nature of the system generates viral escapes in a short time period. Our in-depth analysis of virus populations also represents a template for future studies analyzing virus escape from anti-viral CRISPR transgenics. This is especially important for informing regulation of such actively mutagenic applications of CRISPR-Cas9 technology in agriculture.

**Electronic supplementary material:**

The online version of this article (10.1186/s13059-019-1678-3) contains supplementary material, which is available to authorized users.

## Background

The bacterial CRISPR-Cas9 (clustered, regularly interspaced short palindromic repeats-CRISPR associated 9) gene editing system can be used to engineer resistance to DNA viruses through direct cleavage of the virus genome. Unlike conventional gene editing using CRISPR-Cas9, engineering virus interference requires constitutive and permanent expression of the ribonucleoprotein complex in the host. For example, the CRISPR/Cas9 system has been used to engineer immunity to latent HIV-1 proviruses, hepatitis B viruses, herpes simplex virus, and the human papillomavirus in mammalian cell lines [1]. CRISPR-Cas9 has also been used in the model plants *Arabidopsis thaliana* and *Nicotiana benthamiana* to engineer resistance to single-stranded DNA (ssDNA) geminiviruses [2–4] and a double-stranded DNA (dsDNA) pararetrovirus [5]. However, the degree to which using CRISPR-Cas9 interference to engineer virus-resistance results in the evolution of resistant viruses is unknown. One concern (which has previously been highlighted [6]) might be that planting transgenic, virus-resistant CRISPR-Cas9 plants in the field will impose a selection pressure on viruses, while simultaneously providing viruses with a mechanism (via Cas9-induced mutations) to escape resistance. Transient studies using *Tobacco rattle virus*-based delivery of the CRISPR-Cas9 and gRNA in *Nicotiana benthamiana* suggest that geminiviruses can be repaired by non-homologous end joining (NHEJ) at the target site [7].

Cassava is a tropical staple food crop consumed by more than a billion people. Cassava production in Africa and South Asia can be decimated by cassava mosaic geminiviruses [8]. Biotechnology has proven effective for engineering cassava mosaic virus resistance by using plant endogenous RNA interference (RNAi) pathways to limit the expression of virus genes [9]. However, the fact that plant viruses have developed effective suppressors of RNAi [10], as well as the limited success of RNAi-mediated geminivirus resistance [11], suggests that newer methods of engineering resistance are needed. Using orthogonal systems (i.e., independently evolved systems) like CRISPR-Cas9 to which plant viruses are unlikely to have developed escape mechanisms hence appears particularly attractive. We applied CRISPR-Cas9 to engineer resistance to geminiviruses, specifically the *African cassava mosaic virus* (ACMV) (Begomovirus; *Geminiviridae*) in cassava and investigated the impact of engineering resistance on geminivirus evolution. CRISPR-Cas9 transgenic plants failed to demonstrate effective geminivirus resistance, and we found that the use of CRISPR-Cas9 led to emergence of a novel, conserved mutant virus that cannot be cleaved by CRISPR-Cas9. We urge caution in the application of CRISPR-Cas9 for virus resistance in plants, both in glasshouse and field settings, to avoid inducing the evolution of resistant viruses.

## Results

**Fig. 1:**
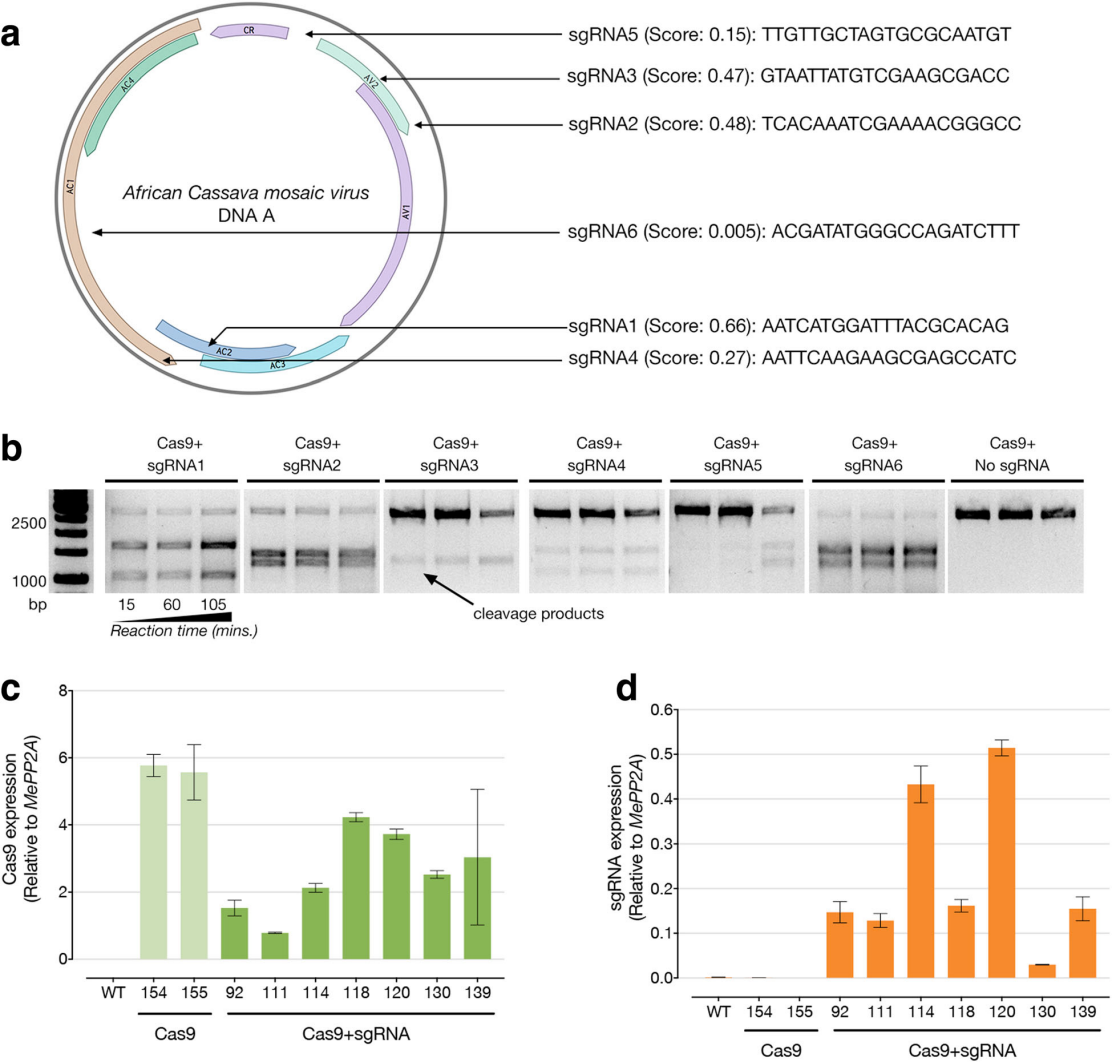
sgRNA design and expression profiling of CRISPR/Cas9 transgenics. **a** Low off-target sgRNAs targeting the DNA A of the *African cassava mosaic virus* and in silico predicted efficiency score (max. efficiency= 1). **b** In vitro cleavage assay for testing the effectiveness of six different sgRNAs against the viral template. **c**, **d** Reverse transcription-quantitative PCR (RT-qPCR) analysis of Cas9 and sgRNA transgene expression respectively. Three independent plants per line were tested

**Fig. 2:**
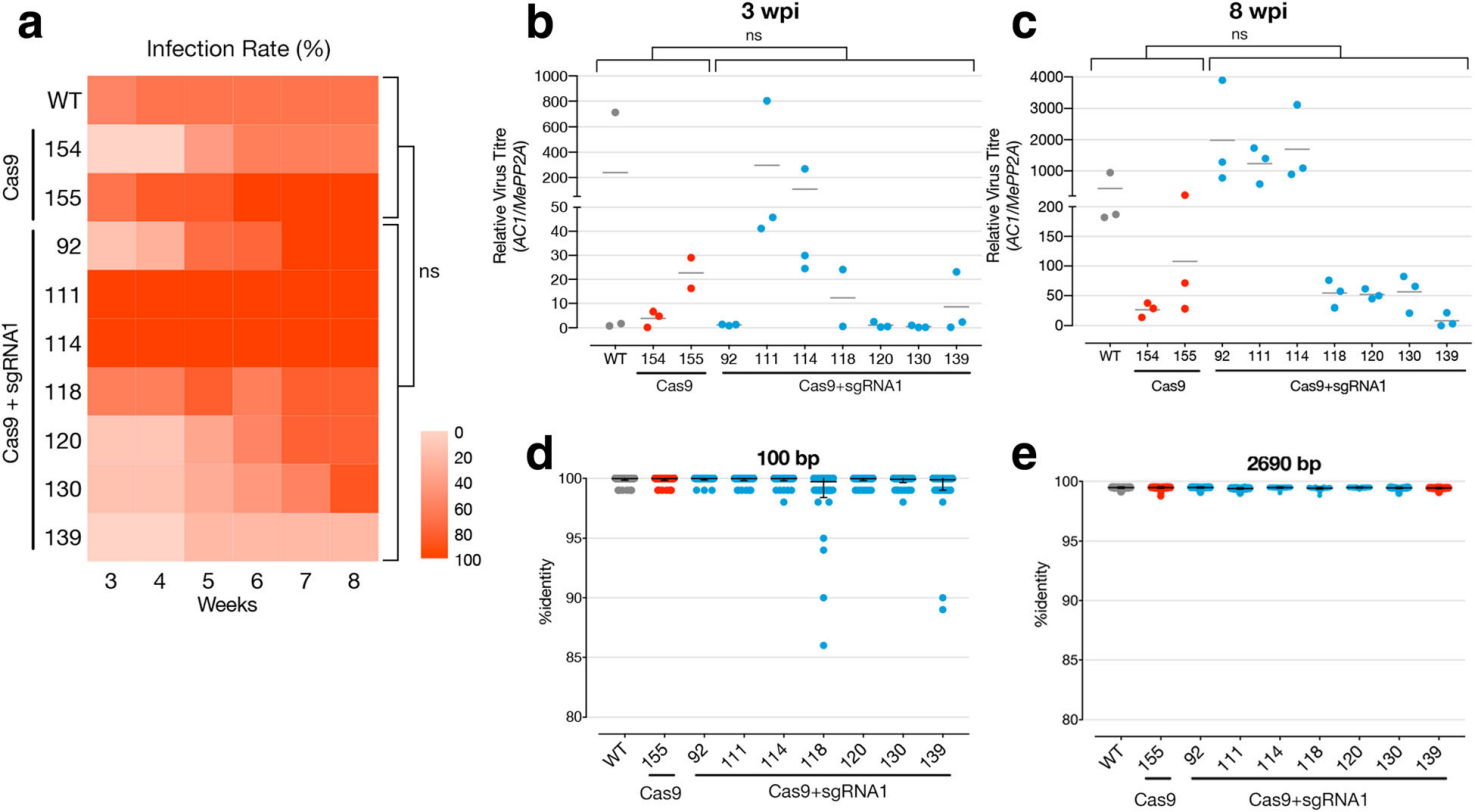
Evaluation of CRISPR/Cas9 expressing transgenics for geminivirus resistance. **a** Infection rates of cassava mosaic disease symptoms on agro-inoculated plants monitored weekly over an 8-week period. A minimum of five independent replicates per transgenic lines were infected. **b** Virus levels in symptomatic plant samples at 3 and **c** 8 weeks post infection (wpi). Levels were measured on three replicated pools of symptomatic leaves from a minimum of five individual plants per line. (ns = no statistical significance observed using Dunn’s multiple comparisons test). **d**, **e** %identity of each virus sequence per line, measured against the reference sequence, across a 100-nt window surrounding the sgRNA site and across almost the entire viral genome respectively

We designed a set of single-guide RNAs (sgRNAs) using a custom algorithm that combines knowledge about potential off-target effects with published software predicting sgRNA template-cleaving efficiency [12]. We chose sgRNA1, which targets both the viral *AC2* gene coding for the multifunctional TrAP protein involved in gene activation, virus pathogenicity, and suppression of gene silencing, and the *AC3* gene coding for the REn protein involved in replication enhancement [13] (Fig. 1a, b). Selected independent transgenic cassava lines (7 Cas9+sgRNA1, 2 control Cas9-only lines, and wild-type controls (WT)) were tested for transgene expression in tissue culture (Fig. 1c, d) and for virus resistance in the greenhouse using an infectious clone of ACMV that was introduced using *Agrobacterium tumefaciens* [9, 14]. No significant differences in disease incidence, symptom severity, or virus titres were found between test and control cassava lines (Table 1) (Fig. 2a–c). This was verified by an additional infection experiment in a subset of lines to account for infection variability (Additional file 1: Table S1).

**Table 1:** Virus infection results

Time post inoculation	Genotype	Line number	Proportion of symptomatic plants	% of symptomatic plants	Mean symptom severity of symptomatic plants (0–3)	% mutant (AC2-H54Q) virus of total
3 weeks	Wild-type	–	5/9	55.5	2	0
	Cas9	154	0/5	60	1.4	0
		155	4/6	66.6	1.7	0
	Cas9+sgRNA	92	1/8	12.5	2	0
		111	5/5	100	2.8	0
		114	6/6	100	2.3	0
		118	3/5	60	1.3	0
		120	1/9	11.1	1	0
		130	1/7	14.3	3	0
		139	0/5	0	0	0
8 weeks	Wild-type	–	6/9	67	1.8	0
	Cas9	154	3/5	60	2.3	0
		155	6/6	100	2.2	0
	Cas9+sgRNA	92	8/8	100	1.9	0
		111	5/5	100	2.6	0
		114	6/6	100	1.8	0
		118	4/5	80	1.7	5.2
		120	7/9	78	1.7	0
		130	6/7	86	2.5	2.2
		139	1/5	20	3	1.6

**Fig. 3:**
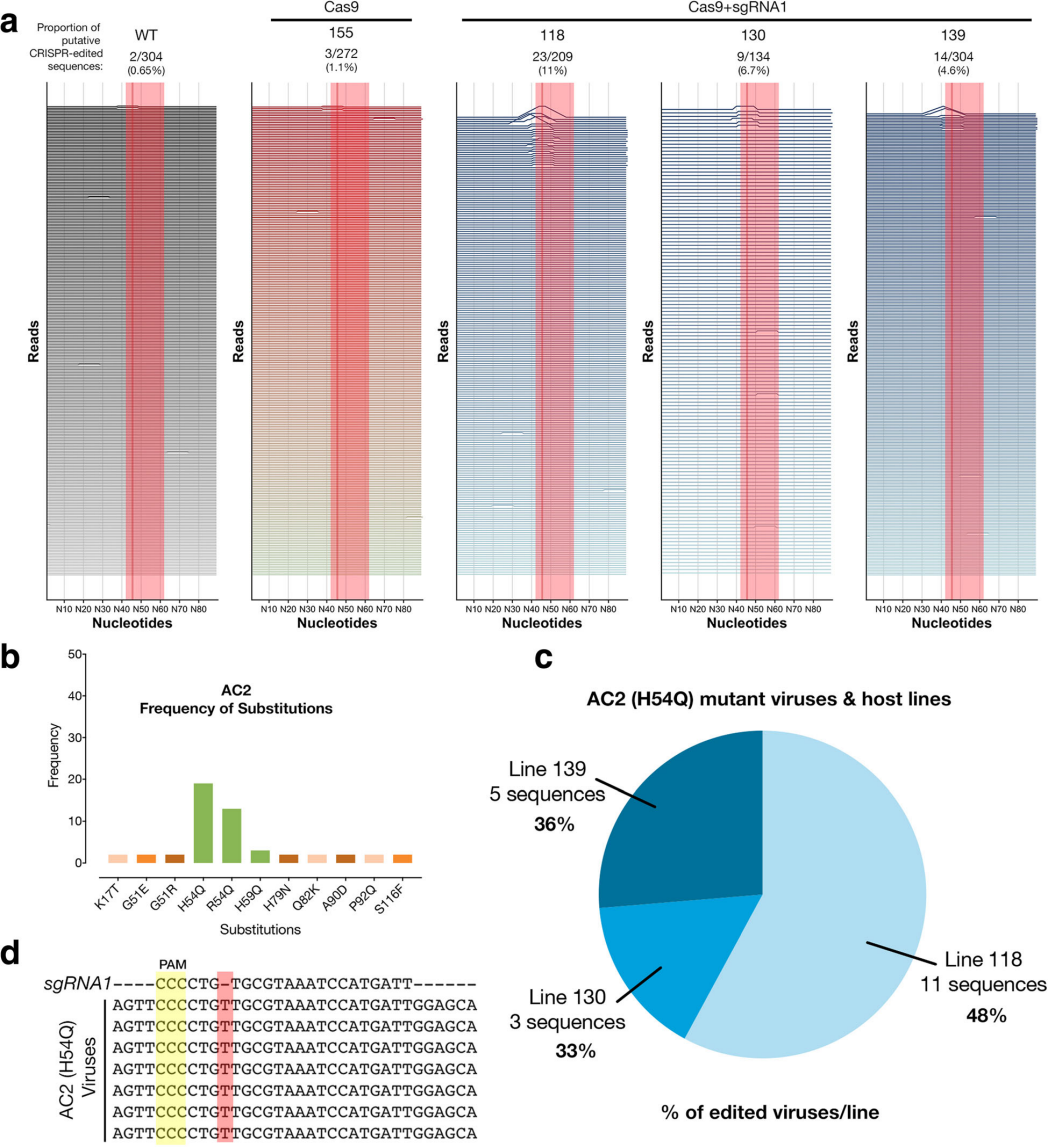
Deep sequencing of CRISPR-edited viruses in cassava transgenics. **a** Analysis of virus sequences from infected plants at 8 weeks post infection. Each horizontal line represents a 90-nt window for each individual virus sequence. Peaks represent edits and are scaled to the %mismatch value of each base pair (see “Methods” for calculation) in a pairwise global alignment with the reference virus sequence. The sgRNA target is indicated by a shaded red rectangle and a dark line represents the putative cut-site. **b** The number of instances of each substitution event in the AC2 protein detected in all the plant lines at 8 wpi. Green bars indicate amino acid substitutions in the sgRNA target region. **c** Distribution of ACMV-AC2 (H54Q) virus variant in different host plant lines. Percentage values represent the proportion of edited viruses in each line which contain the conserved CRISPR-induced mutation. **d** Alignment of some AC2 (H54Q) virus sequences with the sgRNA1 sequence

In order to better understand why we could not detect resistance, we sequenced full-length viral amplicons from pooled infected samples at 3 and 8 weeks post infection (wpi) using single-molecule real-time (SMRT) sequencing with a minimum depth of 100 full-length, high-quality virus genomes per plant (Fig. 3a, Additional file 1: Figure S1). We chose to sequence the whole viral genome in order to check Cas9+sgRNA1-mediated editing of the targeted viral sequences as well as any other targets. A total of 4942 full-length virus genome sequences were analyzed by alignment against the intact wild-type ACMV genome sequence. We detected CRISPR-edited virus sequences in 3 Cas9+sgRNA1 lines at 8 wpi, with line 118 having the highest proportion of edited virus sequences (11%) (Fig. 3a, Fig. 2d,e). We analyzed the AC2 and AC3 genes in depth because these were meant to be edited in Cas9+sgRNA1 lines (Additional file 1: Figures S2 and S3). We found that control lines (WT and Cas9) and 5 of 7 Cas9+sgRNA1 lines had similar proportions of substitution [~ 3%] and indel [~ 3%] events. In Cas9+sgRNA1 line 118, which had the highest proportion of editing events at 11%, there were more indels [14%] than substitutions [3%] (Additional file 1: Figure S2). We analyzed the predicted AC2 and AC3 proteins in all sequenced viruses from all of the cassava lines (density plots, Additional file 1: Figures S2 and S3). These data indicate that the Cas9+sgRNA1 118 line (and to a lesser extent lines 130 and 139) contains viruses with the desired edits, where the AC2 and AC3 open reading frames stop prematurely at the sgRNA target site. We also detected indels that were present in viruses infecting all plants including controls (minor peaks, Additional file 1: Figure S2 and S3, and mismatches, Fig. 3a and Additional file 1: Figure S8). These mutations are likely to represent naturally occurring viral variants in our experiment.

While studying the CRISPR-Cas9 caused amino acid substitutions in our entire dataset, we identified an abundant single-nucleotide insertion in the *AC2* open reading frame (Fig. 3b–e). Interestingly, this conserved mutation was found in three independent Cas9+sgRNA1 lines (but not in any of our three control lines). In each of these lines, this virus variant (named *ACMV-AC2 H54Q*) comprised between 33 and 48% of all mutant viruses per line, which might indicate selection for this conserved mutation (Fig. 1c). In *ACMV-AC2 H54Q*, the single-nucleotide “T” insertion (Fig. 3d) results in a H54Q substitution in the AC2 gene and production of a premature stop codon, reducing the length of the AC2 protein from 136AA to 62AA. However, this mutation also creates a new ORF, which might code for the missing AC2 residues. Thus, in this viral variant (Additional file 1: Data S1), the Cas9-mediated edit at the target site generates two distinct translational units for AC2 (Additional file 1: Figure S2f). Interestingly, the single “T” insertion in *ACMV*-*AC2 H54Q* is in the sgRNA seed sequence. This means that the insertion that is selected for during editing makes *ACMV*-*AC2 H54Q* resistant to further cleavage using sgRNA1, as validated using an in vitro cleavage assay (Additional file 1: Figure S4).

In order to study if the *ACMV-AC2 H54Q* virus variant is capable of independent replication, we generated an infectious agroclone of the mutant virus and inoculated the model plant, *Nicotiana benthamiana*. *N. benthamiana* plants were agro-inoculated (through leaf infiltration) with *ACMV-AC2 H54Q*, *ACMV-WT*, and both *ACMV AC2 H54Q* and *ACMV-WT* virus clones along with negative controls (mock infiltration, as well as the wild-type viral DNA A alone). Plants inoculated with only ACMV-AC2 H54Q (as well as control plants) failed to develop foliar disease symptoms over 4 weeks. Plants co-inoculated with *ACMV-AC2 H54Q* and the WT virus developed severe symptoms, similar to plants inoculated with the WT virus only. We next deep-sequenced full-length viral DNA from new leaves emergent post inoculation (i.e., leaves that could only contain systemically infecting viruses) in order to test if the *ACMV-AC2 H54Q* virus could replicate in the symptomatic plants infected with both the WT and mutant clones. Deep sequencing revealed that the ACMV-AC2 H54Q virus was only detectable in new leaves of plants co-inoculated with mutant and wild-type virus, albeit at lower frequencies of 0.05% compared to ~ 1–5% in the cassava CRISPR transgenics (Additional file 1: Table S2).

## Discussion

In our experiment, CRISPR-Cas9-mediated interference of ACMV in cassava transgenic lines resulted in selection for a conserved, abundant, cleavage-resistant mutant virus among the edited virus genomes. We did not observe a clear disease-resistance phenotype associated with the implementation of a CRISPR-Cas9 sgRNA1virus interference system. Our deep sequencing results also show that the efficiency of CRISPR-mediated virus cleavage was outstripped by the speed of virus replication and that this, in addition to DNA repair of cleaved viruses, resulted in the lack of resistance.

Bacteria and archaea use the CRISPR system to defend against viruses and plasmids, but DNA repair mechanisms differ substantially in bacteria and archaea compared with eukaryotes. Most bacteria lack non-homologous end joining (NHEJ) as a DNA repair mechanism [15], and cleaved phage/plasmid DNA is usually degraded rather than repaired [16, 17]. In eukaryotes NHEJ enables efficient genome editing by effectively repairing cleaved DNA. However, this efficient repair mechanism makes CRISPR-Cas9-mediated virus resistance more prone to evolving mutant viruses. We detected numerous CRISPR-edited viruses whose sequence suggests that dsDNA replicative forms of these viruses have been repaired by NHEJ post-cleavage. Repaired viral genomes have been detected previously in two earlier studies in stable transgenic plants [2, 3], as well as in a study with a viral-vector-delivered CRISPR-Cas9 system in model plants [4] and in studies aimed at engineering virus resistance in mammalian systems [18, 19]. The evolution of stable CRISPR-Cas9 cleavage-resistant viruses in plants has been hypothesized in this journal previously [6], and we now provide confirmation of this in a crop plant. One additional consideration in experiments to engineer virus resistance in eukaryotes is the monitoring period, which in this study was twice as long as in previous studies [2, 3]. We first detected *ACMV-AC2 H54Q* at 8 wpi, suggesting that evolution of resistant viruses may occur later in the infection process.

Our follow-up experiment in the model plant *N. benthamiana* revealed that the replication of the mutant virus detected in the CRISPR transgenics was dependent on the presence of the wild-type virus. This observation suggests that the mutant virus requires the AC2 and AC3 proteins expressed by the wild-type virus. In *N. benthamiana* plants, the mutant virus also accumulated to a much lower degree compared to cassava CRISPR transgenics. This can be explained by the fact that the wild-type *N. benthamiana* plants are unable to select for editing-resistant viruses. Thus, our results indicate that the activity of the CRISPR system at low efficiency (as observed in the cassava transgenics) is sufficient to permit the accumulation of CRISPR-resistant mutant viruses at higher abundances than they would in plants not expressing the abovementioned CRISPR system.

## Conclusions

We found that using CRISPR-Cas9 with a single sgRNA in cassava resulted in the emergence of a CRISPR-resistant virus, with a conserved mutation across three independent transgenic lines. We caution that CRISPR-induced virus evolution could have important implications for field studies using CRISPR-Cas9 to engineer plant virus immunity. The ability of CRISPR systems to trigger the evolution of new viruses would also impact the regulatory mechanisms available for testing and releasing such plants. While regulation of CRISPR-Cas9-edited, but not transgenic, plants has been clarified in the US, Japan and the EU, the regulation of transgenic plants constitutively expressing Cas9 has not yet been considered [20]. We expect that such plants will likely proceed under existing biosafety regulations governing other genetically modified organisms (GMOs). However, our results point to a novel environmental containment consideration for regulating the release of plants constitutively expressing Cas9 and sgRNAs targeting a virus. We highly recommend sequencing the virus population in on-going studies utilizing CRISPR-Cas9 to engineer virus immunity in plants and animals. We also recommend testing whether using more efficient versions of Cas9, targeting multiple virus genes, or using paired Cas9-nickases to delete larger portions of the viral genome can delay the emergence of resistant viruses without enhancing recombination frequencies, which can participate in the emergence of hypervirulent geminivirus isolates [8, 21].

Our findings lead us to conclude that strategies to use CRISPR-Cas9 to engineer virus resistance should be optimized to reduce the emergence of editing-resistant viruses. While the editing-resistant virus in our study is not independently infectious, this mutant may also be an intermediate step towards the development of a truly pathogenic novel virus. One important feature of the CRISPR system in bacterial cells is its ability to adapt to invading DNA/RNA species. Further investigation into the mechanisms that underlie foreign DNA recognition and spacer acquisition by CRISPR systems in prokaryotes may finally result in the engineering of adaptive immunity to viruses in plants. In the meantime, the implementation of technologies with the potential to speed up virus evolution should be carefully assessed as they pose significant biosafety risks.

## Methods

### Plasmid cloning

The GoldenGate/MoClo system [22] was used to construct binary vectors for plant transformation. Level 1 plasmids bearing 35S promoter-driven *hptII* (selectable marker) and plant codon-optimized *cas9-GFP* genes were described in a previous study [23]. T7 promoter-driven sgRNA cassettes for in vitro expression were chemically synthesized (Thermo Fisher) and blunt end cloned into a pJET1.2 vector (Thermo Fisher). The U6 promoter-driven sgRNA1 gene was chemically synthesized and directly cloned into a GoldenGate level 1 vector. Level 1 vectors carrying the *hptII*, *cas9-GFP*, and *sgRNA1* genes were cloned into a level 2 vector via a one-pot reaction to produce binary vectors containing the *hptII* and *cas9* expression cassettes as well as vectors containing the *hptII*, *cas9*, and *sgRNA1* expression cassettes*.*

### sgRNA design

sgRNAs targeting the ACMV DNA A were designed using two parameters, cleavage efficiency and potential off-targets. Only sgRNAs with at least two seed sequence mismatches against the entire 750-Mb cassava genome were selected. First, high-efficiency sgRNAs were obtained using published software from the Broad Institute, MIT [12]. The mismatch search included screening potential sgRNA targets in the cassava reference genome v6.1 (https://phytozome.jgi.doe.gov) with either the canonical NGG and non-canonical NAG protospacer-associated motifs (PAMs) for *SpCas9*. This yielded only 10 sgRNA designs (out of a total of 305 possible sgRNAs) meeting the off-target screening criteria, of which we selected the best six based on efficiency scores and target locations. These six sgRNAs (Additional file 1: Figure S1a) were further tested for effectiveness using an in vitro cleavage assay with purified Cas9/sgRNA complexes to cleave full-length AMCV amplicons (Additional file 1: Figure S1b). We selected sgRNA1 for stable expression in transgenic cassava because it had the best predicted efficiency and performed well in the in vitro cleavage assay.

### In vitro transcription

T7::sgRNA expression cassettes were amplified from their respective pJET1.2 host vectors using primers listed in Additional file 1: Table S3. One microgram of gel-purified linear DNA was used as a template in an in vitro transcription reaction using 100 U of T7 RNA Polymerase (EP0111, Thermo Fisher), 0.1 U inorganic pyrophosphatase (EF0221, Thermo Fisher), 40 U RiboLock RNase Inhibitor (EO0381, Thermo Fisher), and 10 mM NTP mix (R0481, Thermo Fisher) in 1× T7 RNA Polymerase buffer for 16 h at 37 °C. Transcribed sgRNAs were purified by phenol - chloroform extraction.

### In vitro Cas9 cleavage assay

The in vitro Cas9 cleavage assay was performed according to a previously published protocol [24]. ACMV templates for cleavage were purified via PCR amplification from total DNA extracts of infected WT plant tissue using primers listed in Additional file 1: Table S3. The purified GFP-tagged Cas9 endonuclease was kindly provided by Prof Martin Jinek (University of Zurich). Two hundred and fifty nanograms of ACMV template DNA was digested with 1 μM each of purified sgRNA and Cas9 protein. Digestion reactions were stopped at 15, 60, and 105 min. The in vitro cleavage assay to test resistance to the Cas9-sgRNA1 complex was similarly performed using a 409 synthetic dsDNA template (Additional file 1: Data S2) designed from the ACMV-AC2 H54Q sequence.

### Plant transformation and growth conditions

We generated cassava transgenic lines expressing the Cas9 protein together with sgRNA1 (referred to as Cas9+sgRNA1 lines) as well as control lines expressing only the Cas9 protein (referred to as Cas9 lines) using an established *Agrobacterium tumefaciens-*mediated transformation protocol [25]. Twenty transgenic cassava lines were characterized for T-DNA copy number (Additional file 1: Figure S7) and expression of the full-length, 180-kDa GFP-tagged, plant codon-optimized Cas9 protein was verified via Western blotting (Additional file 1: Figure S8). The selected lines expressed both the Cas9 and sgRNA transgenes at varying levels (Additional file 1: Figure S1c,d).

In vitro transformed cassava plantlets were grown at 28 °C in a 16-h photoperiod and sub-cultured at 4-week intervals in CBM media (1× Murashige-Skoog medium, 2% sucrose, 2 μM copper sulfate, 0.3% gelrite, pH 5.8). Thirty-day-old plantlets were transferred to soil and grown in glasshouse conditions (14 h photoperiod, 60% relative humidity, day/night temperatures, 26 °C/17 °C).

### Southern blotting for T-DNA integration analysis

Total DNA was extracted from leaves harvested from 4-week-old in vitro grown plants using a modified CTAB (cetyl trimethylammonium bromide) protocol [26]. The same leaf samples were used for Western blots and reverse transcription-quantitative PCR (RT-qPCR) analysis. Ten micrograms of total DNA was restriction digested with 20 U of HindIII (Thermo Fisher) in an overnight reaction. The digested DNA was separated on a 0.8% agarose-TAE gel and transferred overnight onto a positively charged nylon membrane (Roti-Nylon Plus, Carl Roth). A 700-bp probe against the *hptII* gene was PCR amplified from the binary vector and labeled with [α-32P] dCTP using the Prime-A-Gene kit (Promega). The nylon membrane was treated with PerfectHyb Plus Hybridization Buffer (Sigma-Aldrich) for 30 min followed by hybridization together with the radio-labeled probe. Blots were developed using a Typhoon FLA 7500 imaging system (GE Healthcare Life Sciences).

### Western blotting

Crude protein extracts were prepared by incubating ground leaf tissue in 5% SDS, 125 mM Tris-HCL (pH 6.8), 15% glycerol buffer with 1× EDTA-free Complete Protease Inhibitor (Roche) for 20 min at room temperature. Samples were centrifuged at 4 °C for 10 min to clear debris. Fifty micrograms of total protein extract was electrophoresed (after a 1:1 dilution with a bromophenol blue solution) on a pre-cast Novex 4–20% Tris-Glycine Midi gel (Thermo Fisher) and transferred to a PVDF membrane (Carl Roth) using a TransBlot Cell (Bio-Rad) system according to the manufacturer’s instructions. Membranes were blocked using 5% milk and 1× Tris buffered saline, 0.1% Tween20 (1XTBS-T) solution for 1 h at room temperature. The blocked membrane was incubated in a primary blotting solution (1XTBS-T) with SpCas9 monoclonal (mouse) antibody (Diagenode) at a 1:2500 dilution and a polyclonal Actin (Rabbit) antibody (Agrisera) at a 1:1000 dilution for 1 h at room temperature. After three 5-min washes with 1XTBS-T, the membrane was incubated with a secondary blotting solution containing IRDye 800CW Goat anti-Mouse IgG and IRDye 680RD Goat anti-Rabbit IgG antibodies at a 1:5000 dilution each. Blots were imaged using the LICOR Odyssey CLx fluorescence imaging system. A PageRuler Prestained protein ladder (Thermo Fisher) was used for size estimation.

### Reverse transcription-quantitative PCR

1.5 μg of total RNA extract from leaf samples was DNase 1 treated and reverse transcribed with random hexamer primers using the Revert-Aid First strand cDNA Synthesis Kit (Thermo Fisher) according to the manufacturer’s instructions. Quantitative PCR was carried out with the fast SYBR Green dye for 40 cycles on a Lightcycler 480 instrument (Roche). Relative quantitation was performed using the *MePP2A* reference gene using the primers listed in Additional file 1: Table S3. Results of transgene-expression quantitation using RT-qPCR are presented in Additional file 1: Figure S1 c, d)

### Virus inoculation and symptom monitoring in cassava

*Agrobacterium tumefaciens* (strain LBA4404) cells carrying infectious clones of ACMV-NOg DNA A and DNA B [9, 14] were cultured for 48 h at 28 °C in 5 mL YEB broth (5 g/L tryptone, 1 g/L yeast extract, 5 g/L nutrient broth, 5 g/L sucrose, 2 mM MgSO4) supplemented with antibiotics (25 mg/L rifampicin, 100 mg/L streptomycin, and 50 mg/L kanamycin). Two milliliters of the starter cultures was then individually added to 200 mL YEB medium with antibiotics and incubated overnight at 28 °C to an OD_600_ of 0.6–1. Cells were pelleted by centrifuging at 5000×*g* for 10 min, then re-suspended in 5 mL inoculation medium (10 mM MES pH 5.6, 10 mM MgCl_2_, 0.25 mM acetosyringone) and incubated for 2 h at room temperature with shaking. DNA A and DNA B cultures at an OD_600_ of 2.0 were mixed in equal proportions prior to inoculation.

For inoculation, all leaves save the top leaf were removed from 6-week-old cassava plantlets (65 plants in total). The stem and axillary buds were pricked prior to dipping the plantlets in Agrobacterium solution for 10 s and subsequently covered in a Plexiglas box for 3 days. A minimum of five inoculated plants per line were monitored for symptom incidence and severity over a period of 8 weeks. Symptoms in the top three leaves were scored weekly from 3 to 8 weeks post inoculation (wpi) on a scale of 0–3 as depicted in Additional file 1: Figure S7. The first emerging leaf from each plant was harvested 3 wpi. The top three leaves were harvested from each plant at 8 wpi.

### Virus cloning and inoculation in *N. benthamiana*

A 582-bp fragment of the *ACMV-AC2 H54Q* virus flanked by naturally occurring EcoRI and AflII restriction sites was chemically synthesized (Thermo Fisher). The original agroclone of the wild-type virus was digested with EcoRI and AflII, and the newly synthesized and digested fragment was ligated in place, without any scarring. The clone was sequence verified by Sanger sequencing and electroporated into electrocompetent *A. tumefaciens* (strain LBA4404) cells. Agrobacterium cultures were grown and prepared for inoculation as described above (with 150 mM acetosyringone). Cells were infiltrated using a Softject 1-mL syringe in a single fully expanded leaf. Six *N. benthamiana* plants per test construct (and three plants per control construct) were infiltrated and monitored over 4 weeks of growth at 22 °C, 12 h light. After 4 weeks, the four top-most fully expanded leaves from each plant (not including the infiltrated leaf) were harvested for DNA extraction and virus sequencing.

### Quantitation of virus titres

Virus quantitation was performed by RT- qPCR on 10 ng of total DNA extracts using ACMV DNA A-specific primers and *MePP2A* genomic DNA reference primers as listed in Additional file 1: Table S2. Symptomatic leaves were harvested in three separate pools for each plant line (in the absence of symptomatic leaves, asymptomatic leaves were used). Three technical replicates were used per pooled sample. Results are shown in Fig. 2b, c.

### Single molecule real-time sequencing of viral amplicons

Full-length viral amplicons from selected plant lines were prepared using target-specific primers tailed with universal sequences (Additional file 1: Table S3) according to the protocol provided by Pacific Biosciences Inc. Equal amounts of each amplicon were used as a template in a second PCR using the Barcoded Universal Primers provided by Pacific Biosciences Inc. (Barcodes used per sample are listed in Additional file 1: Table S4). The standard SMRTBell library construction protocol was used to prepare a pooled, barcoded, sequencing library. Sequencing was performed using a standard MagBead loading protocol on a PacBio RSII instrument. Polymerase reads were processed into barcode separated subreads by primary analysis on the instrument. The resulting subreads [27] were processed using a standard circular consensus sequencing (CCS) analysis using SMRTPipe with a configuration file specifying a minimum predicted quality of 99.9 and a minimum length of 2600 bp. For the *N. benthamiana* experiment, an equimolar pool of amplified viral DNA from each replicate plant was pooled to prepare two sequencing pools: *ACMV-AC2 H54Q*+*ACMV-WT*, and *ACMV-WT* alone. Each pool was amplified using separate Barcoded Universal Primers and sequenced as described above.

### Sequence analysis

Sequences representing a near full-length region (2692 bp) of the ACMV DNA A genome as well as a 100-bp region surrounding the sgRNA target site were extracted from each ROI in order to maintain identical start and end sequence positions in each viral amplicon sequence read. Each resulting sequence was pairwise aligned (global alignment using NEEDLE parameters) against its corresponding reference ACMV-NOg DNA A sequence (GenBank: AJ427910). Pairwise alignment scores were assigned to each nucleotide as the sequence mismatch percentage of a 10-nucleotide window surrounding it [28]. The resulting per-base score (*y*-axis) along each sequence read was plotted using the ggplot and ggjoy packages in R to produce Fig. 1a and Additional file 1: Figure S3. Total pairwise identity scores were used to create Additional file 1: Figure S2d,e. Background mismatches resulting from either sequencing errors or viral variants were found in all lines, including controls. We also failed to find any conserved edits on viruses infecting Cas9+sgRNA1 lines (and not control lines), indicating the absence of an off-target on the virus genome (Additional file 1: Figure S8). This was expected because the seed sequence of sgRNA1 does not have a close match to another site on the viral genome.

For the *N. benthamiana* experiment, SMRT sequencing raw reads were error-corrected using the CCS pipeline at a threshold of 99.5 predicted accuracy. Resulting full-length virus sequences were searched with the mutant sgRNA target site to count the occurrence of mutant viruses.

## Additional file

Additional file 1: **Table S1.** Virus infection results (confirmation experiment). **Table S2.** Proportion of *ACMV-AC2 H54Q* viruses detected by deep-sequencing in *N. benthamiana.* **Table S3.** Primer sequences. **Table S4.** Sequencing Barcodes. **Figure S1.** Analysis of virus sequences from infected plants at (a) 3 and (b) 8 weeks post infection. **Figure S2.** Analysis of viral proteins from edited and control populations obtained by single molecule amplicon sequencing at 3 weeks post infection. **Figure S3.** Analysis of viral proteins from edited and control populations obtained by single molecule amplicon sequencing at 8 weeks post infection. **Figure S4.** In vitro cleavage assay of the ACMV-AC2 H54Q mutant. **Figure S5.** Southern blot analysis for number of T-DNA integration events per plant line. * **Figure S6.** (a) Western blots for Cas9-GFP expression. (b) Raw blot images acquired using an Odyssey CLX imager for anti-Cas9 and anti-Actin probing of protein extracts from Cas9+sgRNA1 lines. (c) Raw blot images for probing Cas9 lines protein extracts with anti-Cas9 and anti-Actin antibodies. **Figure S7.** Symptom scoring scale. **Figure S8.** Analysis of full-length virus sequences from infected plants at 8 weeks post infection. (DOCX 6747 kb)

## Abbreviations

ACMV: African cassava mosaic virus
CRISPR-Cas9: Clustered, regularly interspaced short palindromic repeats-CRISPR associated 9
dsDNA: Double-stranded DNA
NHEJ: Non-homologous end joining
PAM: Protospacer-associated motif
sgRNA: Single-guide RNA/synthetic guide RNA
ssDNA: Single-stranded DNA
wpi: Weeks post infection
