# Supplementary material for: Linking CRISPR-Cas9 interference in cassava to the evolution of editing-resistant geminiviruses
Source: Genome Biol. 2019 Apr 25;20:80. doi: 10.1186/s13059-019-1678-3 (PMC6482539; doi:10.1186/s13059-019-1678-3)
Supplement: Supplementary file 1 — Table S1. Virus infection results (confirmation experiment). Table S2. Proportion of ACMV-AC2 H54Q viruses detected by deep-sequencing in N. benthamiana. Table S3. Primer sequences. Table S4. Sequencing Barcodes. Figure S1. Analysis of virus sequences from infected plants at (a) 3 and (b) 8 weeks post infection. Figure S2. Analysis of viral proteins from edited and control populations obtained by single molecule amplicon sequencing at 3 weeks post infection. Figure S3. Analysis of viral proteins from edited and control populations obtained by single molecule amplicon sequencing at 8 weeks post infection. Figure S4. In vitro cleavage assay of the ACMV-AC2 H54Q mutant. Figure S5. Southern blot analysis for number of T-DNA integration events per plant line. * Figure S6. (a) Western blots for Cas9-GFP expression. (b) Raw blot images acquired using an Odyssey CLX imager for anti-Cas9 and anti-Actin probing of protein extracts from Cas9+sgRNA1 lines. (c) Raw blot images for probing Cas9 lines protein extracts with anti-Cas9 and anti-Actin antibodies. Figure S7. Symptom scoring scale. Figure S8. Analysis of full-length virus sequences from infected plants at 8 weeks post infection. (DOCX 6747 kb) [file 13059_2019_1678_MOESM1_ESM.docx]

## Table S1: Virus infection results (confirmation experiment)

| **Genotype** | **Line Number** | **Proportion of Symptomatic Plants** | **Percentage of infection** |
| --- | --- | --- | --- |
| Wild-type |  | 12/12 | 100% |
| Cas9 - 154 | 154 | 2/2 | 100% |
| Cas9 - 155 | 155 | 16/16 | 100% |
| Cas9+sgRNA | 111 | 18/20 | 90% |
| Cas9+sgRNA | 114 | 3/3 | 100% |
| Cas9+sgRNA | 118 | 1/1 | 100% |
| Cas9+sgRNA | 120 | 5/5 | 100% |
| Cas9+sgRNA | 130 | 10/10 | 100% |
| Cas9+sgRNA | 139 | 8/8 | 100% |

Symptomatic plants observed at 9 weeks post-inoculation. Plants were inoculated twice at 2 weeks interval.

## Table S2: Proportion of *ACMV-AC2 H54Q* viruses detected by deep-sequencing in *N. benthamiana*

| Inoculum | Proportion of plants with symptoms | PCR detection of virus | % mutant virus of total  (#mutant virus reads/#total viral reads)* |
| --- | --- | --- | --- |
| Mock | 0/3 | 0/3 | - |
| ACMV-WT | 6/6 | 6/6 | 0% (0/4437) |
| ACMV-WT DNA A | 0/3 | 0/3 | - |
| ACMV-AC2 H54Q | 0/6 | 0/6 | - |
| ACMV-AC2 H54Q+ACMV-WT | 6/6 | 6/6 | 0.05% (5/9249) |

## Table S3: Primer sequences

| Primer Name | Sequence | Use |
| --- | --- | --- |
| mePP2A_genomic_F | CGC TGT GGA AAT ATG GCA TCA | Cassava qPCR reference gene |
| mePP2A_genomic_R | CTG GCT CAA ACT GCA GGA TCA A |  |
| CMV_qPCR_F | GGT CCT GGA TTG CAG AGG AAG ATA GTG GG | Cassava geminiviral DNA quantitation |
| CMV_qPCR_R | GGT ACA ACG TCA TTG ATG ACG TCG ATC CC |  |
| SMRT_UNI_CMV_F | GCA GTC GAA CAT GTA GCT GAC TCA GGT CAC GGA ATG CCA CCT TTA ATT TGA | Target specific primers tailed with SMRT Universal sequences |
| SMRT_UNI_CMV_R | TGG ATC ACT TGT GCA AGC ATC ACA TCG TAG TAT CTT CCT CTG CAA TCC |  |
| sgRNA-RTq-F | GAATCATGGATTTACGCACAGGTTTTAGAG | RT-qPCR for sgRNA expression |
| sgRNA-RTq-R | CGACTCGGTGCCACTTTTTCAAGTTG |  |

## Table S4: Sequencing Barcodes

| Barcode | Line | Time-point  (weeks after infection) |
| --- | --- | --- |
| 1 | WT | 3 |
| 9 | 155 | 3 |
| 49 | 139 | 3 |
| 41 | 130 | 3 |
| 33 | 118 | 3 |
| 25 | 114 | 3 |
| 17 | 111 | 3 |
| 57 | WT | 8 |
| 73 | 92 | 8 |
| 65 | 155 | 8 |
| 34 | 139 | 8 |
| 26 | 130 | 8 |
| 18 | 120 | 8 |
| 10 | 118 | 8 |
| 2 | 114 | 8 |
| 89 | 111 | 8 |
| 81 | 110 | 8 |

# Supplementary Figures


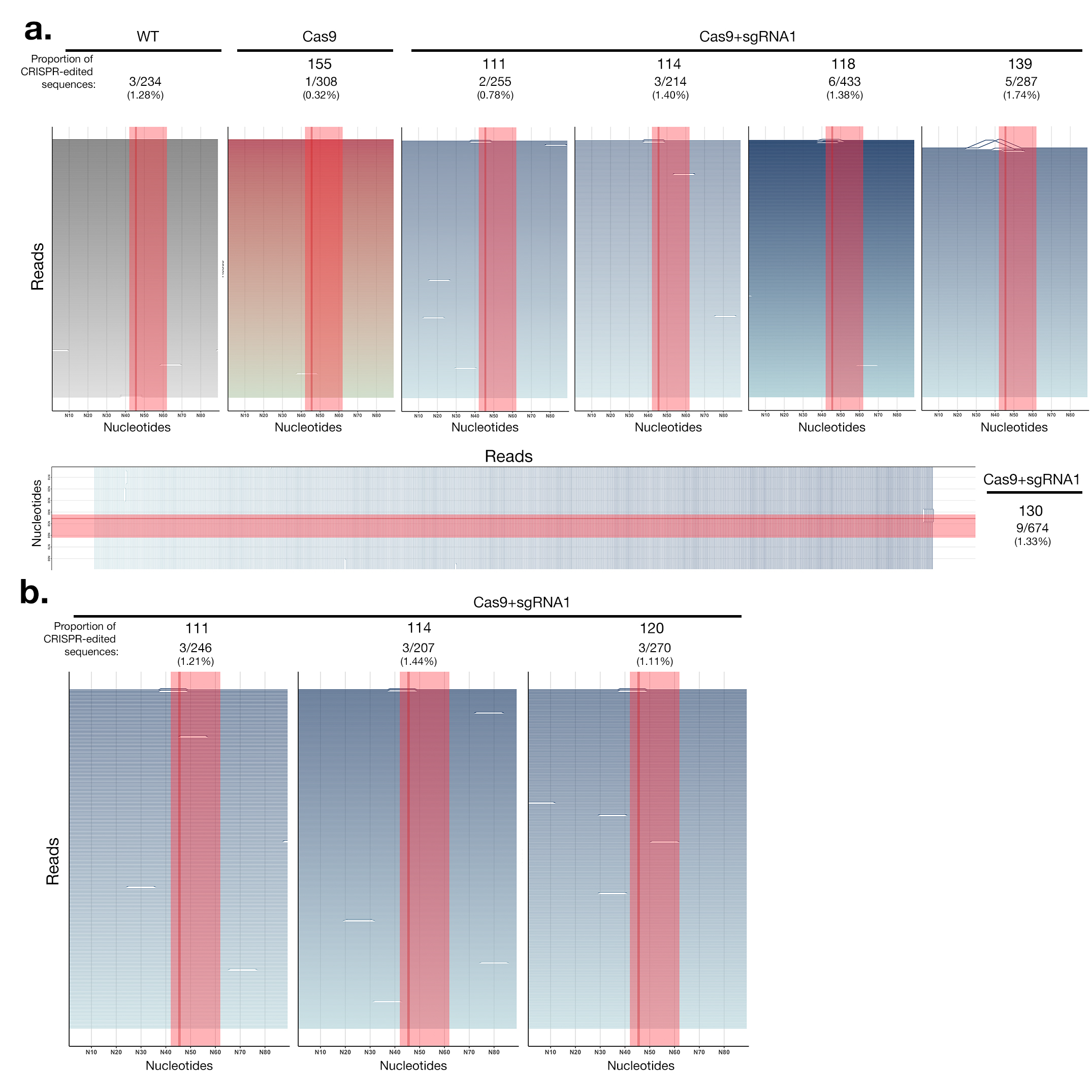


**Fig. S1:** Analysis of virus sequences from infected plants at **(a)** 3 and **(b)** 8 weeks post infection. Each horizontal line represents a 90bp window for each individual virus sequence. Peaks represent edits and are scaled to the %mismatch value of each base-pair (see Methods for calculation) in a pairwise global alignment with the reference virus sequence. The sgRNA target is indicated by a shaded red rectangle and a dark line represents the putative cut-site.

**
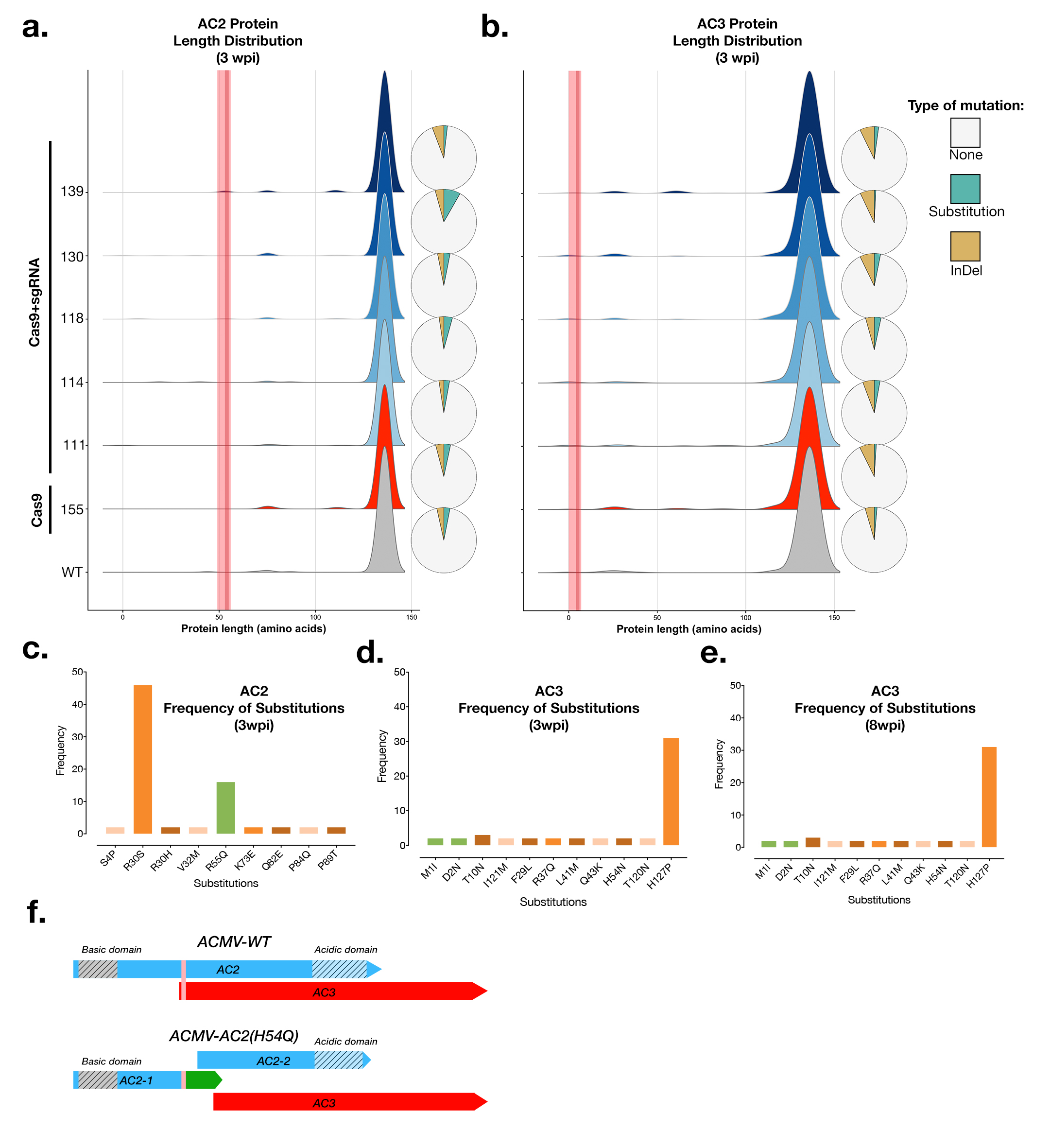
**

**Fig. S2:** Analysis of viral proteins from edited and control populations obtained by single molecule amplicon sequencing at 3 weeks post infection. **(a)** Density plots show the frequency distribution of virus sequences with different AC2 protein lengths. Pie charts show the type of mutations detected as a percentage of total virus sequences detected in each line. Thus, minor peaks in the distribution show the reduction in protein length caused by the indels (yellow) detected in viruses infecting each line. Red bars show the location of the sgRNA target (light red) and the predicted cleavage site (dark red). **(b)** The number of instances of each substitution event in the AC2 protein detected in all the plant lines. Green bars indicate mutations in the sgRNA target region. **(c)** & **(d)** the same for the AC3 protein at 3 wpi, and **(e)** at 8 wpi. **(f)** Scheme of the AC2 and AC3 open reading frames in the wild-type and mutant (AC2-H54Q) ACMV virus (vertical pink bar shows the sgRNA target region).


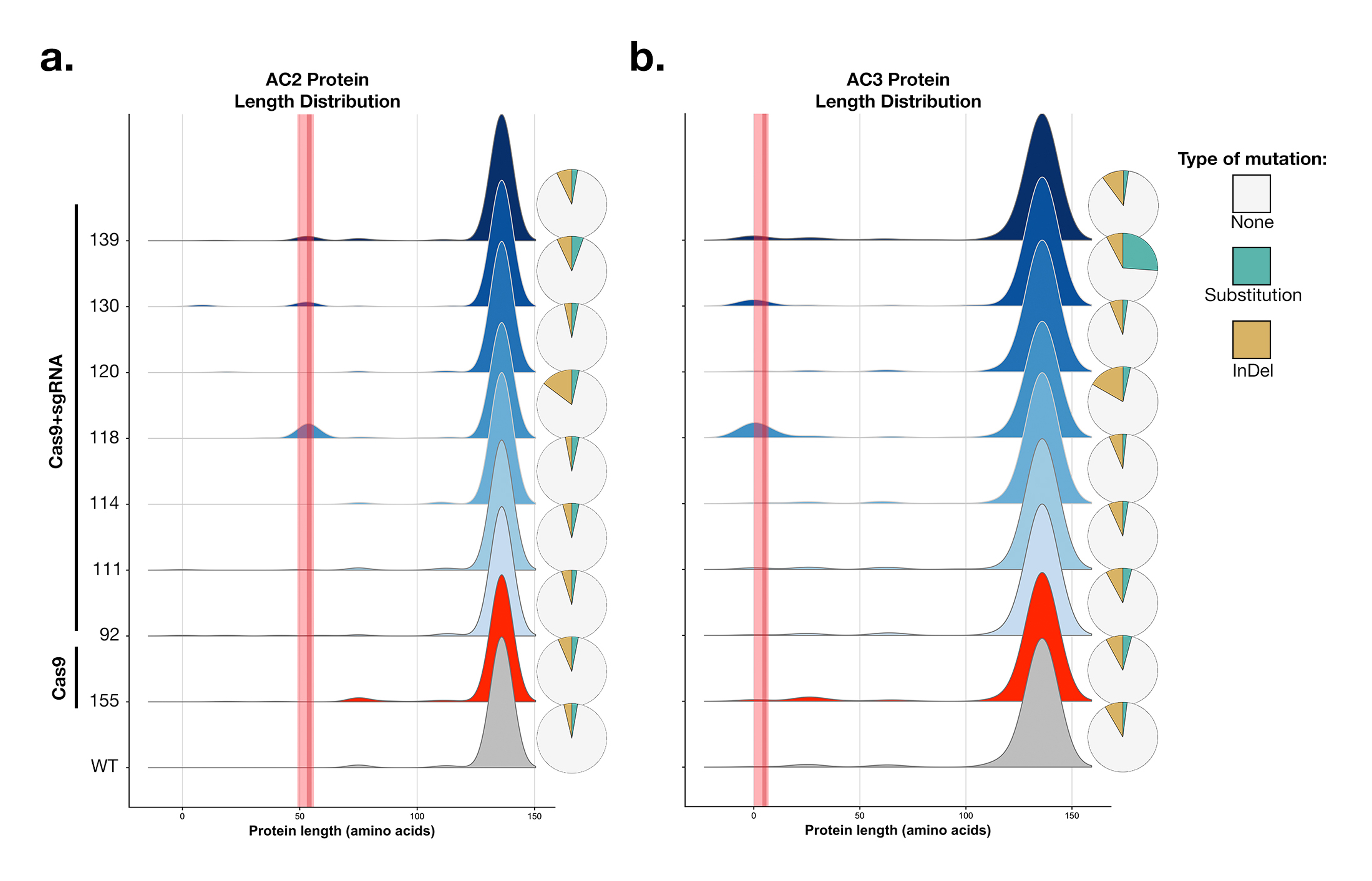


**Fig. S3:** Analysis of viral proteins from edited and control populations obtained by single molecule amplicon sequencing at 8 weeks post infection. **(a)** Density plots show the frequency distribution of virus sequences with different AC2 protein lengths. Pie charts show the type of mutations detected as a percentage of total virus sequences detected in each line. Thus, minor peaks in the distribution show the reduction in protein length caused by the indels (yellow) detected in viruses infecting each line. Red bars show the location of the sgRNA target (light red) and the predicted cleavage site (dark red). **(b)** the same for the AC3 protein at 8 wpi.


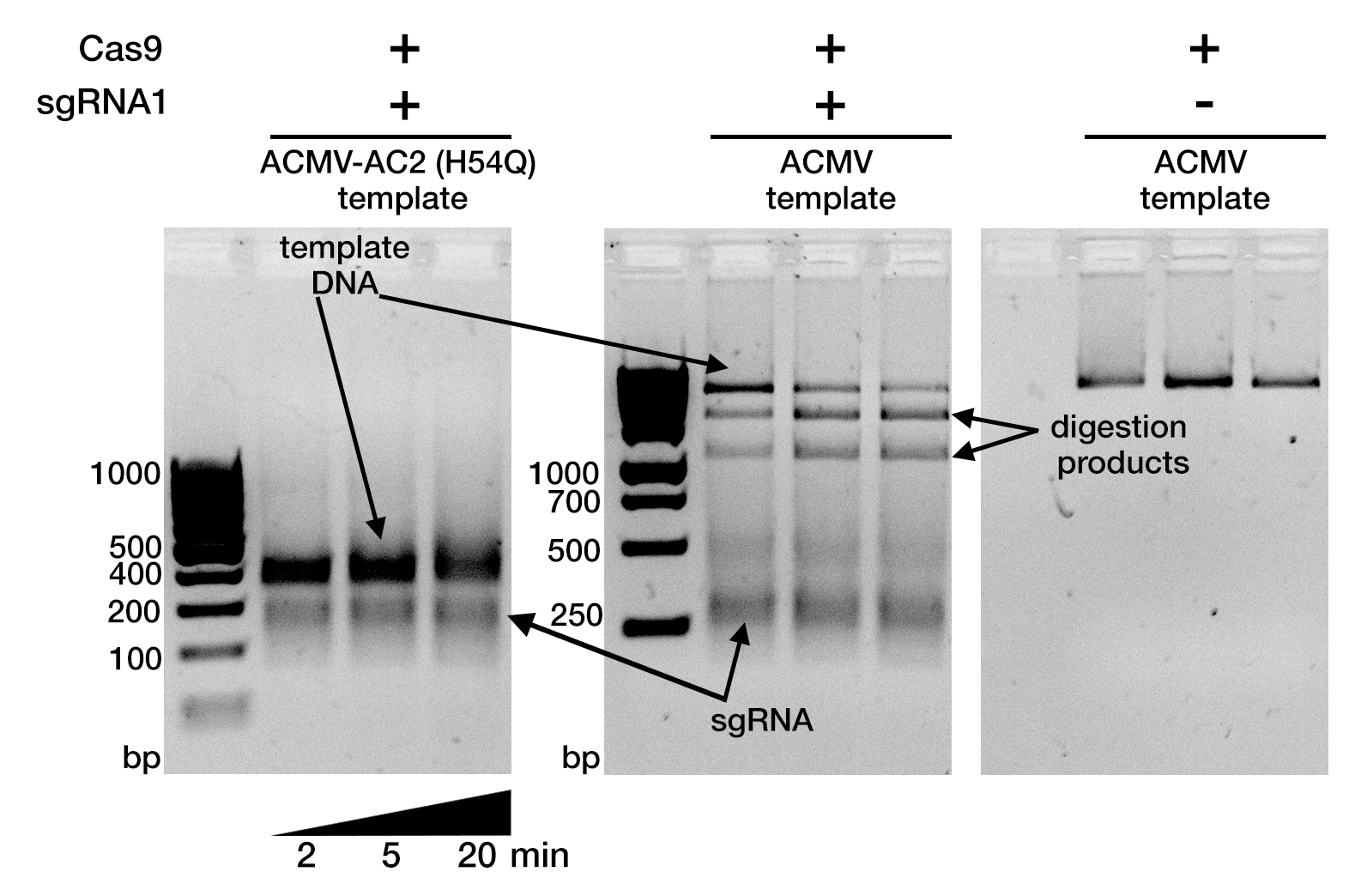


**Fig. S4:** *In vitro* cleavage assay of the ACMV-AC2 H54Q mutant. Treatment of a 409 bp ACMV-AC2 (H54Q) dsDNA template with purified Cas9-GFP and sgRNA1 fails to cleave the ACMV-AC2 (H54Q) amplicon (the expected cleavage products are 248 and 161bp long). The full-length ACMV template used in Fig. 1b is used as positive control and its Cas9-sgRNA1 digestion generates two fragments that are 1696 bp and 1074 bp long.

**
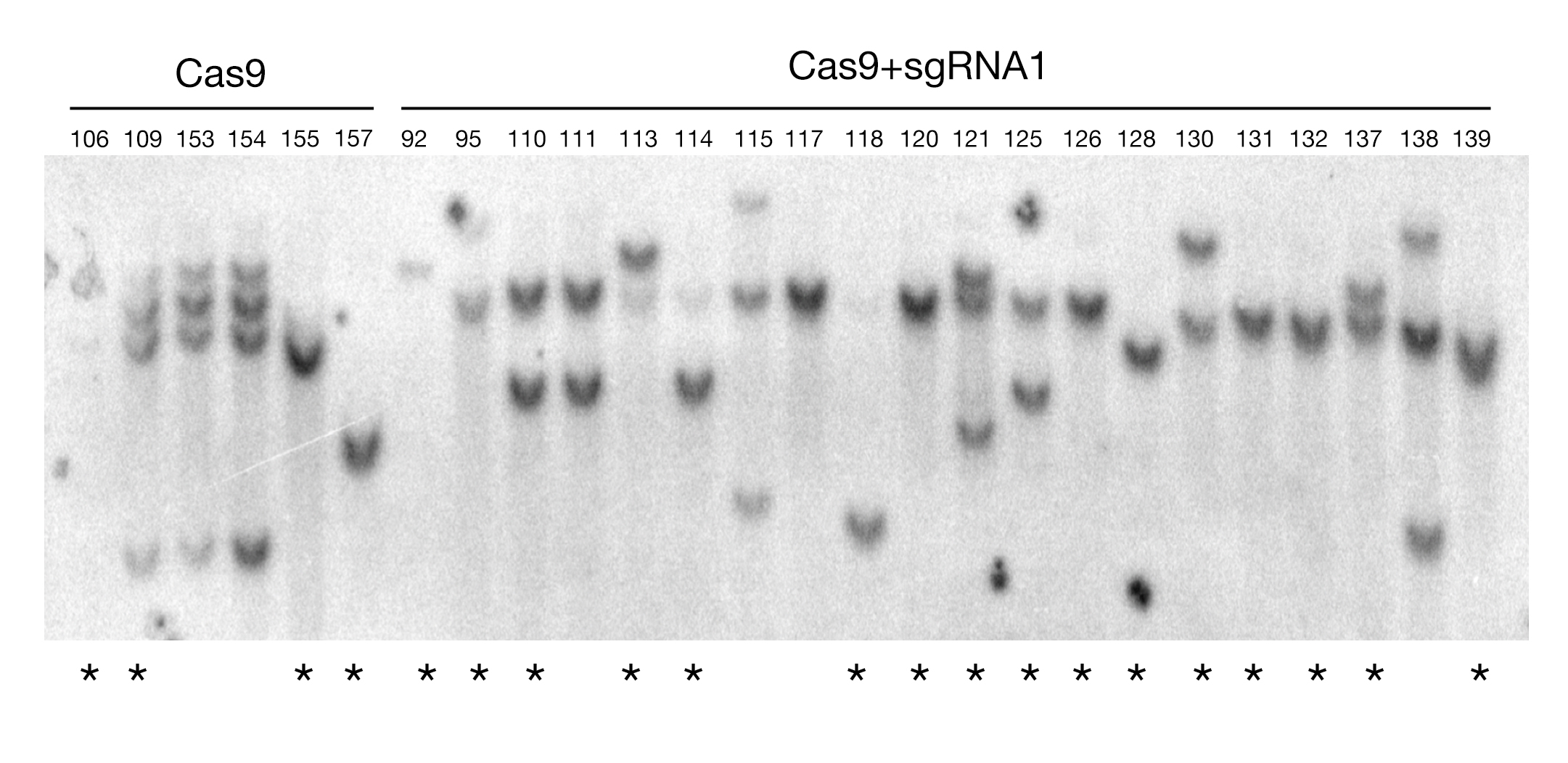
**

**
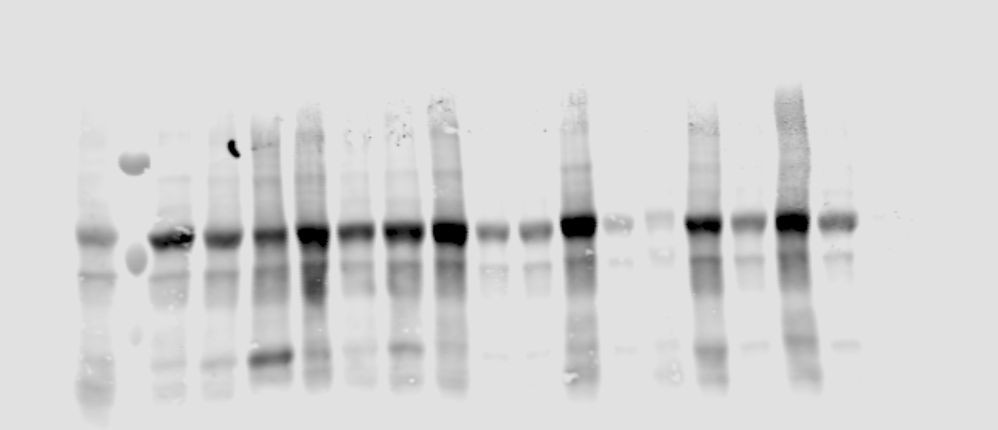

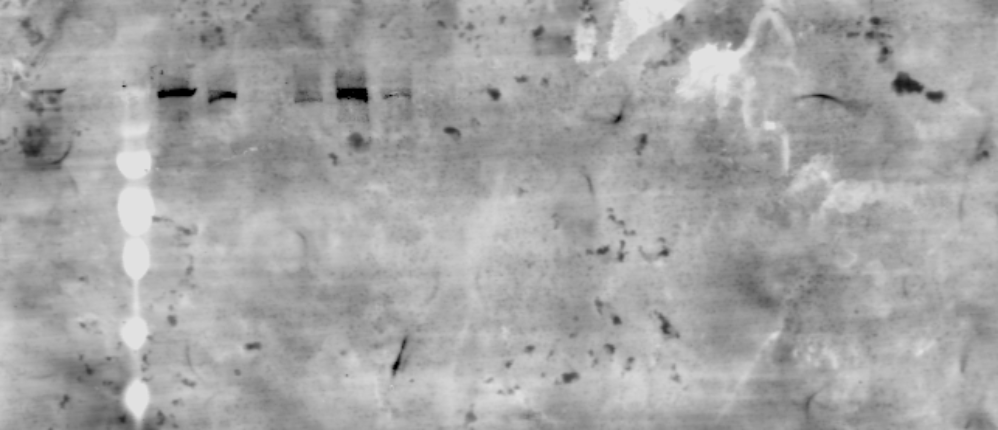

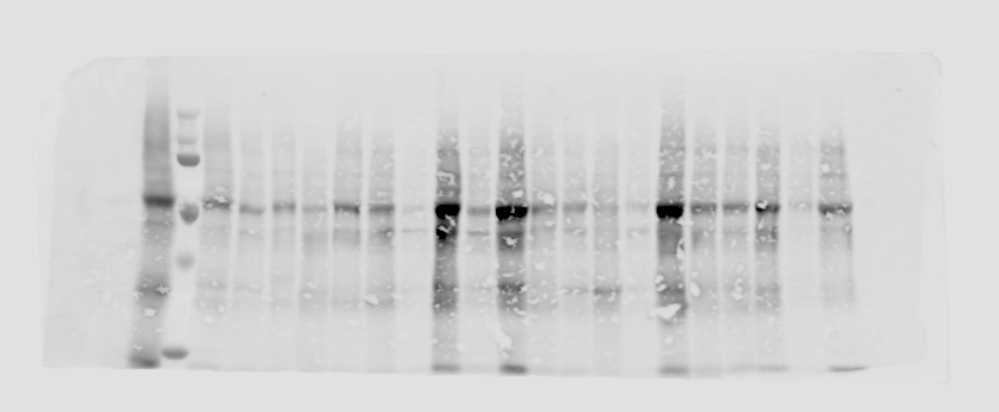

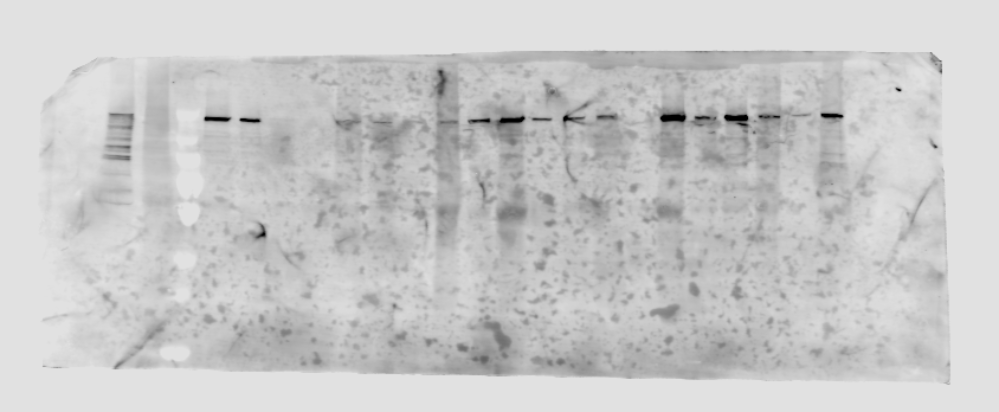

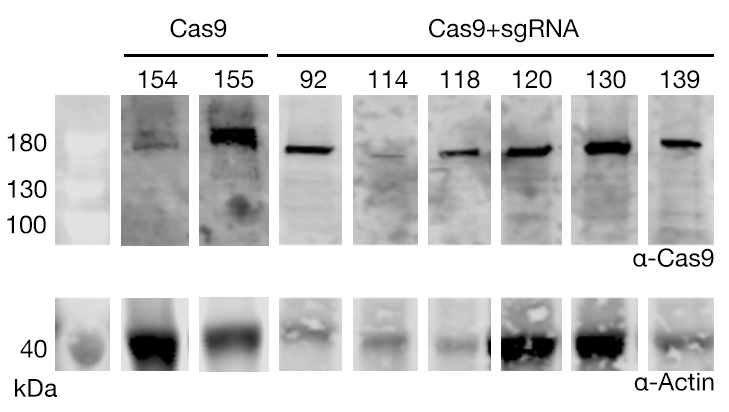
Supplementary Figure 5:** Southern blot analysis for number of T-DNA integration events per plant line. * indicates independent lines.

Cas9+sgRNA1 lines

Cas9 lines

**α-Actin**

**α-Cas9**

**c.**

**b.**

**α-Actin**

**α-Cas9**

**a.**

**Fig. S6: (a)**Western blots for Cas9-GFP expression. **(b)** Raw blot images acquired using an Odyssey CLX imager for anti-Cas9 and anti-Actin probing of protein extracts from Cas9+sgRNA1 lines. **(c)** Raw blot images for probing Cas9 lines protein extracts with anti-Cas9 and anti-Actin antibodies.

**
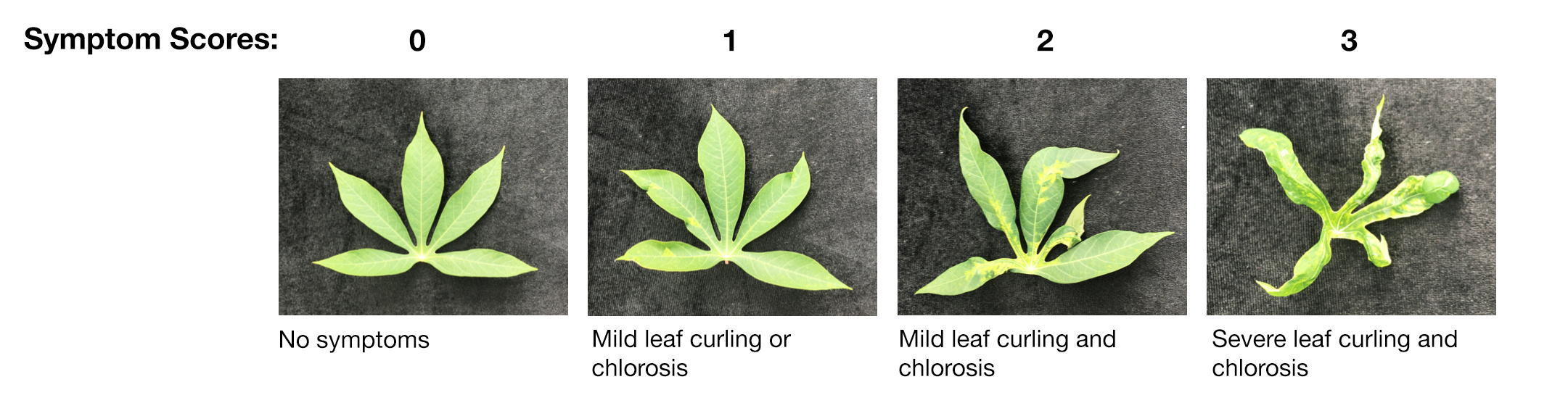
**

**Fig. S7:** Symptom scoring scale.


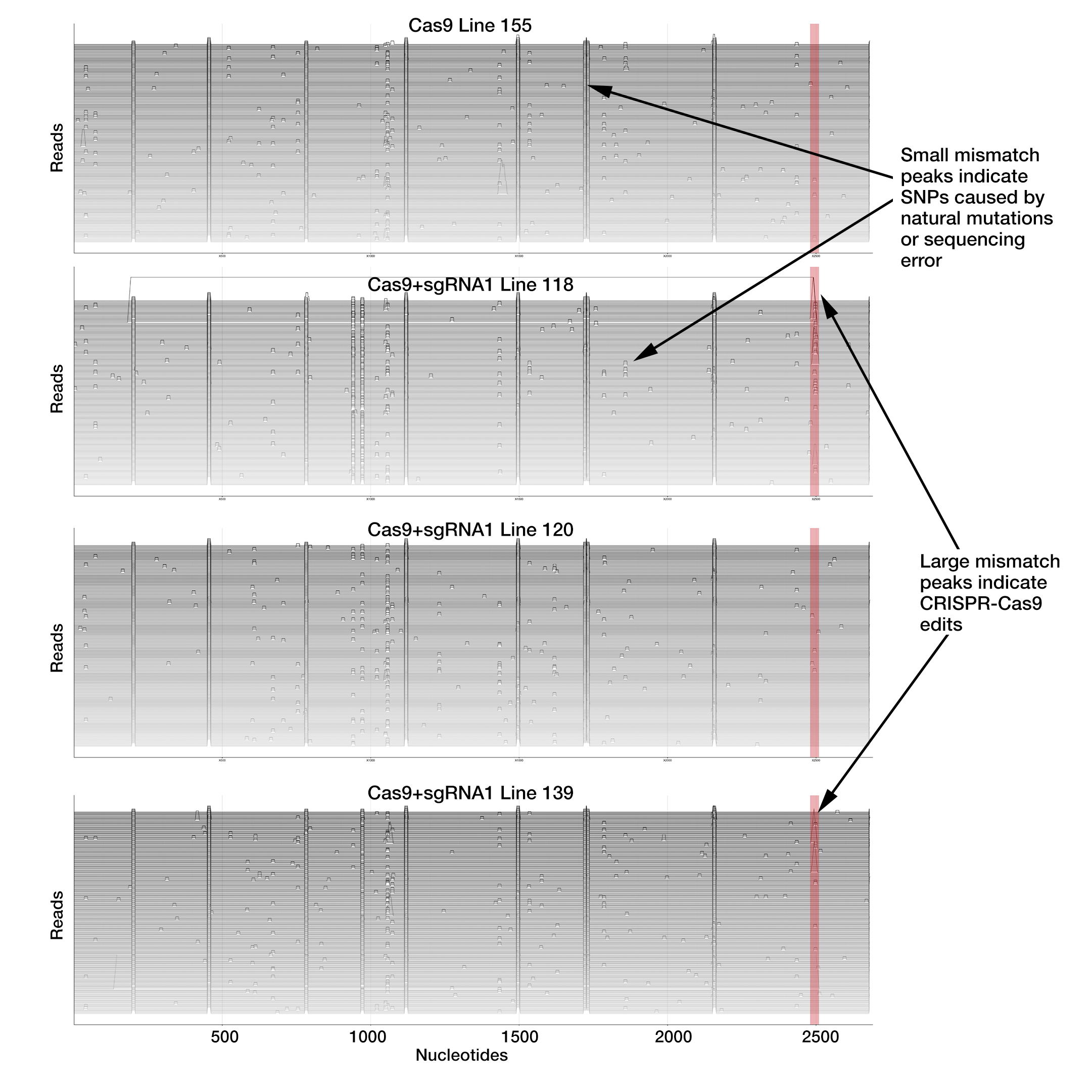


**Fig. S8:** Analysis of full-length virus sequences from infected plants at 8 weeks post infection. Each horizontal line represents a near full-length individual virus sequence (2690bp). Peaks represent edits and are scaled to the %mismatch value of each base-pair (see Methods for calculation) in a pairwise global alignment with the reference virus sequence. The sgRNA target is indicated by a shaded red rectangle. The absence of CRISPR-Cas9 edits (large, tall peaks) in non-target regions indicates a lack of significant off-target cleavage.

# Data S1

LOCUS ACMV-NOg:AC2(H54Q) 2692 bp ds-DNA linear 14-AUG-2017

DEFINITION .

FEATURES Location/Qualifiers

CDS complement(411..962)

/label="AC4"

/ApEinfo_revcolor=#75c6a9

/ApEinfo_fwdcolor=#75c6a9

CDS 1338..2114

/label="AV1"

/ApEinfo_revcolor=#f58a5e

/ApEinfo_fwdcolor=#f58a5e

CDS complement(2470..2664)

/label="AC2del"

/ApEinfo_revcolor=#c7b0e3

/ApEinfo_fwdcolor=#c7b0e3

CDS 1178..1519

/label="AV2"

/ApEinfo_revcolor=#ffef86

/ApEinfo_fwdcolor=#ffef86

CDS complement(1..867)

/label="AC1"

/ApEinfo_revcolor=#b1ff67

/ApEinfo_fwdcolor=#b1ff67

CDS complement(2256..2516)

/label="AC3_new"

/ApEinfo_revcolor=#c6c9d1

/ApEinfo_fwdcolor=#c6c9d1

CDS complement(2111..2473)

/label="new_ORF"

/ApEinfo_revcolor=#f58a5e

/ApEinfo_fwdcolor=#f58a5e

ORIGIN

1 GGGTTTCCCG TATTTCGTGT TGGACTGCCA GTCCCTCTGG GACCCCATGA ATTCTTTAAA

61 GTGCTTTAGG TAGTGGGGAT CGACGTCATC AATGACGTTG TACCAGGCAG CATTATTGAA

121 GACCTTTGGA CTAAGGTCCA GGTGTCCACA CAGGTAATTG TGTGGGCCTA AAGATCTGGC

181 CCATATCGTC TTCCCTGTTC TGCTATCACC TTCTATTACA ATACTATTGG GTCTCCATGG

241 CCGCGCAGCG GAATCCCTAA CATTATCAGC GACCCATTCT TCAATTTCAA CAGGAACTTG

301 GTCAAAGGAA GAACATGGGA AGGGAGAAAC ATAAGGAGCT GGTGGCTCCT GGAAAATCCT

361 ATCTAAATTA CTATTGAGAT TATGAAATTG AAGTACAAAG TCCTTTGGGA CTAATTCCCT

421 AATGACATTA AGAGCTTCTG ACTTACTGCC GCTGTTAAGC GCTTTGGCGT AAGCATCATT

481 CGCTGATTGT TGACCGCCTC TAGCAGATCG TCCATCGATC TGAAATTGTC CCCATTCGAC

541 GGTGTCTCCG TCCTTATCCA GATAGGACTT GACATCTGAG CTTGATTTGG CACCTTGAAT

601 GTTGGGGTGG AAATTGGTGC TACAGGTTGG GTGTACACAA TCGAAGAGAC GATTGTTCGT

661 AATCGTGATT TTCCCCTCGA ATTGGATGAG GGCATGCAAG TGAGGTTCCC CATTCTGATG

721 CAGCTCTCTA CAGATTTTAA TGAACTTAGG GTTTGATGGG AGAGATAGTG TTTGAATGAA

781 TGACAGCAGG TGTTCTTTGG GTATAGAACA CTTTGGGTAT GTGAGAAAGA CATTCTTGGC

841 TTGAACTCTA AAACGCGGAG TTCGCATGTT GACCAAGTCA ATTGGAGACA CTCAACTAGA

901 GACACTCTTG AGCATCTCCT CCTGTTAATT GGAGACATTA TATAGTTGTC TCTAAATGGC

961 ATTCTTGTAA TAAGTTCAAC TTTAATTTGA ATTAAAAGGC TCAAAAGGCG CAGAACACCC

1021 AAGGGGCCAA CCGTATAATA TTACCGGTTG GCCCCGCCCC CCTTTAAACG TGGTCCCCGC

1081 GCACTACTTA TGTCGGCCAA TCATGCTGTA GCTTTAAAGG TTAGTTATTA GTGGTGGACC

1141 ACTATATACT TACAGGCGAA GTTGTTGCTA GTGCGCAATG TGGGATCCAC TGGTGAATGA

1201 GTTTCCAGAC TCGGTGCATG GGCTTAGGTG TATGCTTGCA ATTAAATATT TGCAGGCCTT

1261 AGAGGATACA TACGAGCCCA GTACTTTGGG CCACGATTTG GTTAGAGATC TAGTCTCAGT

1321 TATCAGGGCT CGTAATTATG TCGAAGCGAC CAGGAGATAT CATCATTTCC ACTCCAGGCT

1381 CGAAGGTTCG TCGAAGGCTG AACTTCGACA GCCCATACAG GAACCGTGCT ACTGCCCCCA

1441 CTGTCCACGT CACAAATCGA AAACGGGCCT GGATAAACAG GCCCATGTAC AGAAAGCCCA

1501 TGATGTACAG GATGTATAGA AGCCCAGACA TACCTAGGGG CTGTGAAGGC CCATGTAAGG

1561 TCCAGTCGTA TGAGCAGAGG GATGATGTGA AGCACCTTGG TATCTGTAAG GTGATTAGTG

1621 ATGTGACACG TGGGCCTGGG CTGACACACA GGGTCGGAAA GAGGTTTTGT ATCAAGTCCA

1681 TTTACATACT TGGTAAGATC TGGATGGATG AAAATATTAA GAAGCAGAAT CACACTAATA

1741 ATGTGATGTT TTATCTGCTT AGGGATAGAA GGCCTTATGG CAATGCGCCC CAAGACTTTG

1801 GGCAGATATT TAATATGTTT GATAATGAGC CCAGTACTGC AACAATTAAG AACGATTTGA

1861 GGGATAGGTT TCAGGTATTG AGGAAATTTC ATGCCACTGT TATTGGTGGT CCATCTGGCA

1921 TGAAGGAGCA GGCTTTGGTT AAAAGGTTTT ACAGGTTGAA TCATCACGTG ACATATAATC

1981 ATCAGGAGGC AGGGAAGTAT GAGAATCACA CAGAGAATGC TTTGCTTCTG TACATGGCAT

2041 GTACTCATGC CTCCAATCCT GTATATGCTA CGTTGAAAAT ACGTATATAC TTCTATGACA

2101 GTATTGGCAA TTAATAAACA TTGAATTTTA TTTCATGAGT CAACTGACAC TCAATAGTTT

2161 TTTCAATTAC ATTGAACAAA ACATGATCAG CAGCTCTAAT GACATCGTTA ATTGAGATAA

2221 CACCTATATT ATCCAAGTAT TTAAGTACTT GGTATCTAAA GACCCTTAAG AAAAGACCAG

2281 TCTGAGGCCG TAAGGTCGTC CAGATCCTGA AGTTGAGAAA ACATTTGTGA ATCCCCAGCT

2341 CCTTCCTCAG GTTGTGATTG AATCGAACCT GGACTGTTAT GATGTCCTGG TTCAGCAGGA

2401 ATGGTCGTTG TTGGTGCCTG GTGATTGTGA AATACAGGGG ATTGTTTATT TCCCAGGTAT

2461 ACACGCCATT CATTGCTTGA GGAGCAGTGA TGAGTTCCCC TGTTGCGTAA ATCCATGATT

2521 GGAGCAGTTG ATATGGAGGT AATATGAACA GCCACAGACA AGATCCACTC TCCTACGCCG

2581 GATGGCTCGC TTCTTGAATT GTCTGTGACT GACTTTGATT GGAACCTGAG TAGAGTGGTT

2641 CTGTGAGGGT GATGAAGATT GCATTCTTTA ATGCCCAGGC CTTTAGCGCC TC

//

# Data S2

>ACMV-AC2(H54Q)_cleavage_template (sgRNA1 cleavage site marked)

ATGCAATCTTCATCACCCTCACAGAACCACTCTACTCAGGTTCCAATCAAAGTCAGTCACAGACAATTCAAGAAGCGAGCCATCCGGCGTAGGAGAGTGGATCTTGTCTGTGGCTGTTCATATTACCTCCATATCAACTGCTCCAATCATGGATTTACGCA|ACAGGGGAACTCATCACTGCTCCTCAAGCAATGAATGGCGTGTATACCTGGGAAATAAACAATCCCCTGTATTTCACAATCACCAGGCACCAACAACGACCATTCCTGCTGAACCAGGACATCATAACAGTCCAGGTTCGATTCAATCACAACCTGAGGAAGGAGCTGGGGATTCACAAATGTTTTCTCAACTTCAGGATCTGGACGACCTTACGGCCTCAGACTGGTCTTTTCTTAAGGGTCTTTAG
